# Changes in the Effective Connectivity of the Social Brain When Making Inferences About Close Others vs. the Self

**DOI:** 10.3389/fnhum.2020.00151

**Published:** 2020-04-29

**Authors:** Sofia Esménio, José Miguel Soares, Patrícia Oliveira-Silva, Óscar F. Gonçalves, Karl Friston, Joana Fernandes Coutinho

**Affiliations:** ^1^Psychological Neuroscience Laboratory, CIPsi, School of Psychology, University of Minho, Campus Gualtar, Braga, Portugal; ^2^Life and Health Sciences Research Institute (ICVS), School of Medicine, University of Minho, Campus Gualtar, Braga, Portugal; ^3^ICVS/3B’s—PT Government Associate Laboratory, Braga/Guimarães, Portugal; ^4^Clinical Academic Center, Braga, Portugal; ^5^Human Neurobehavioral Laboratory, CEDH—Research Centre for Human Development, Faculdade de Educação e Psicologia, Universidade Católica Portuguesa, Porto, Portugal; ^6^Spaulding Center for Neuromodulation, Spaulding Rehabilitation Hospital, Harvard Medical School, Boston, MA, United States; ^7^Wellcome Centre for Human Neuroimaging, University College London, London, United Kingdom

**Keywords:** social cognition, self and other, brain network, effective connectivity, DCM, PEB

## Abstract

Previous research showed that the ability to make inferences about our own and other’s mental states rely on common brain pathways; particularly in the case of close relationships (e.g., romantic relationships). Despite the evidence for shared neural representations of self and others, less is known about the distributed processing within these common neural networks, particularly whether there are specific patterns of internode communication when focusing on other vs. self. This study aimed to characterize context-sensitive coupling among social brain regions involved in self and other understanding. Participants underwent an fMRI while watching emotional video vignettes of their romantic partner and elaborated on their partner’s (other-condition) or on their own experience (self-condition). We used dynamic causal modeling (DCM) to quantify the associated changes in effective connectivity (EC) in a network of brain regions involved in social cognition including the temporoparietal junction (TPJ), the posterior cingulate (PCC)/precuneus and middle temporal gyrus (MTG). DCM revealed that: the PCC plays a central coordination role within this network, the bilateral MTG receives driving inputs from other nodes suggesting that social information is first processed in language comprehension regions; the right TPJ evidenced a selective increase in its sensitivity when focusing on the other’s experience, relative to focusing on oneself.

## Introduction

Social neuroscience research has shown that when trying to understand another’s emotional and mental states, we rely on psychological processes and brain systems similar to those that we use to understand our internal states (e.g., Lamm et al., 2016). Indeed, similar brain networks are recruited when processing self and other’s internal states in both affective (Singer et al., 2004; Jackson et al., 2005; Lamm et al., 2011), and cognitive tasks (Ochsner et al., 2004; Mitchell et al., 2006; Lombardo et al., 2010). Consistent with these findings—and supporting the idea that inferring or understanding oneself and others are only “semi-independent skills” (Dimaggio et al., 2008)—recent evidence suggests that enhancing the capacity to understand our thoughts and feelings increases the ability to infer those of others (Böckler et al., 2017).

According to simulation theories of social cognition, the closer the other is to oneself, the more likely we are to ground inferences about them on knowledge about oneself (Aron et al., 1991; Gallese and Goldman, 1998; Adolphs, 2002; Goldman, 2006; Gallese, 2014). Therefore, close relationships (such as romantic partners) suggest themselves as a relevant context to characterize the intimate relationship between self and other processing. A previous study, in which participants were presented video-vignettes of their romantic partners, confirmed a significant overlap between the functional anatomy of self and other processing, implicating brain regions associated with both socio-affective and socio-cognitive systems (Esménio et al., 2019).

Despite the evidence for shared neural representations of self and others (e.g., Lombardo et al., 2010), less is known about how information flows within these common neural networks, specifically, are there specific patterns of internode connectivity when focusing on other vs. on the self? The objective of the present study was to characterize the information flow among social brain regions involved in self and other understanding. We, therefore, analyzed the effective connectivity (EC) in a network of brain regions involved in social cognition that included the bilateral temporoparietal junction (TPJ), the posterior cingulate (PCC)/precuneus (prec) and the bilateral middle temporal gyrus (MTG; e.g., Van Overwalle, 2009; Bzdok et al., 2012; Schilbach et al., 2012; Schurz et al., 2014; Alcalá-López et al., 2018). These particular brain regions had previously been shown to be engaged by the experimental paradigm used in the present study (Esménio et al., 2019).

The role of each of these regions for social processing is well-documented. For example, the MTG is primarily involved in language-related processes, such as semantic processing and speech perception (Spreng et al., 2009; Amft et al., 2015), story and narrative comprehension (Mar, 2011), memory processing; particularly, autobiographical memory (Spreng et al., 2009) and emotional processing (Schilbach et al., 2012). The PCC/prec is involved in a wide range of highly integrated tasks (Cavanna and Trimble, 2006), is associated with both self-related processes including self-representation and self-reflection (Cavanna and Trimble, 2006; Johnson et al., 2006), consciousness (Vogt and Laureys, 2005; Northoff et al., 2006), future thinking and prospective memory (Christoff and Gordon, 2008) and other related processes, such as the theory of mind (TOM) or mentalizing (Saxe and Powell, 2006; Bzdok et al., 2012; Schurz et al., 2014); narrative comprehension (Mar, 2011) as well as in empathic and forgivability judgments (Farrow et al., 2001; Ochsner et al., 2004).

Finally, the TPJ—a key social brain region—is traditionally associated with social-cognitive processes such as visual perspective-taking and mental inference/TOM (Saxe and Kanwisher, 2003; Aichhorn et al., 2006; Van Overwalle, 2009; Ramsey et al., 2013; Schurz et al., 2014; Kanske et al., 2015). Importantly, whereas the left TPJ (LTPJ) seems to be mainly involved in language processes (Binder et al., 2009) and intention detection (Berthoz et al., 2002), the right TPJ (RTPJ) has been mostly associated with self-awareness (Decety and Lamm, 2007), and differentiation between self and other perspectives (Santiesteban et al., 2012a; Steinbeis, 2015). Indeed, the RTPJ has been shown to play a major role during TOM (Saxe and Kanwisher, 2003), especially when a difference in perspective exists between self and other (Aichhorn et al., 2006; Sommer et al., 2007; Santiesteban et al., 2012a). In short, the right TPJ appears to play a crucial and context-sensitive role in functional integration, when making inferences about others, relative to self.

As previously mentioned despite the converging findings supporting the involvement of these brain areas in social understanding, a functional integration among these regions has not been established. In particular, the changes in coupling between (i.e., extrinsic connectivity) and within (i.e., intrinsic connectivity) these regions that underwrite differential processing during inferences about self and others are largely unknown. This research requires the use of analytical approaches such as dynamic causal modeling (DCM) that allow us to characterize the causal relationships between the brain nodes of a network.

DCM is a generative model-based Bayesian approach that infers EC within networks of distributed brain regions (Razi and Friston, 2016), in which neuronal responses were measured either with fMRI (Friston et al., 2003; Friston, 2009) or electromagnetic responses such as EEG and MEG (David et al., 2006; Kiebel et al., 2008). Particularly in fMRI, DCM combines a dynamic forward neuronal model of how cortical regions interact and influence each other with a detailed biophysical hemodynamic model that transforms neuronal activity into the measured response (i.e., blood oxygen level response—BOLD; Buxton and Frank, 1997; Friston et al., 2003; Stephan and Friston, 2010; Daunizeau et al., 2011). This combination of an* a priori* biologically plausible neural network model with the measured BOLD response, makes it possible to infer the information flow in terms of directed intrinsic and extrinsic EC; namely the effect that one neuronal system has on another (Friston, 2009; Daunizeau et al., 2011).

As the method of choice for modeling causal interactions in neuroimaging data, task-related DCM has been extensively applied to study a wide variety of processes in both clinical and non-clinical populations. Some examples include DCM studies on speech perception (Osnes et al., 2011), motor functioning (Minkova et al., 2015), language and motor rehabilitation post-stroke (Rehme et al., 2011; Kiran et al., 2015), attention (Fairhall et al., 2009), executive function in major depression (Schlösser et al., 2008), spatial and lexical processing (Deng et al., 2012), response inhibition and working memory in Schizophrenia (Allen et al., 2010; Zhang et al., 2013), inter-hemispheric integration in Alzheimer’s disease (Rytsar et al., 2011).

In this study, we used DCM combined with Parametric Empirical Bayes (PEB) and Bayesian model reduction (BMR) to examine the changes in EC associated with focusing on the partner’s internal states, in comparison to focusing on one’s own experiences (Friston et al., 2016). Technically, this entails inverting a fully connected model for each subject, using subject-specific posteriors over the model parameters to estimate group means (using PEB) and then removing redundant parameters (using BMR). In our application, the key parameters of interest were changes in intrinsic and extrinsic connectivity that model the context-sensitive changes in coupling or information flow due to focus on other, vs. self. Following on previous work using this experimental paradigm (Esménio et al., 2019), our primary hypothesis was that focusing on the partner (other conditions) would increase the sensitivity of the RTPJ to its afferents from other nodes within the social brain network under study.

## Materials and Methods

### Participants

Forty-two participants—in a monogamous romantic relationship for at least 1 year—were enrolled in this study. Before any procedure, all participants were screened on the telephone for an assessment concerning inclusion/exclusion criteria. Inclusion criteria included: (1) the absence of any diagnosed neuropsychiatric or neurodegenerative disorder; (2) absence, in the past year, of a dependency/abuse of alcohol or drugs; (3) ability to attend the MRI session; and (4) age between 20 and 50 years. In the final sample, all the participants were Caucasian, right-handed and the age of the participants ranged from 23 to 40 years old (*M* = 31.17, *SD* = 4.76; for men: *M* = 32.13, *SD* = 4.89, for women: *M* = 30.22, *SD* = 4.50). The majority of participants had college degrees (68%). The mean duration of the relationship was 7.78 years (*SD* = 4.76; range = 1–15 years). 33.3% were married couples, 36.7% were living together, and 30.0% were in dating relationships. Additionally, 38% of the couples had children.

The study complied with the principles expressed in the Declaration of Helsinki (with the amendment of Tokyo 1975, Venice 1983, Hong Kong 1989, Somerset West 1996, Edinburgh 2000) and was approved by the local University Institutional Review Board. At the beginning of this study, the procedure was explained to the participants who provided informed written consent.

### Experimental Task

Each participant watched a set of video-vignettes of his/her romantic partner expressing emotional contents, and was asked to, while watching, either focus on his/her own experience (self condition) or his/her partner’s experience (other condition). These video-vignettes of 20 s duration containing the expression of positive and negative emotional contents were extracted from a previously video-recorded interaction task performed in the lab, where participants shared things that they either liked (positive videos) or disliked (negative videos) about their partner (more details regarding the interaction task can be found in Coutinho et al., 2017, 2018).

The final fMRI task comprised two blocks (self and other), each containing three different conditions: positive trials (*N* = 8), negative trials (*N* = 8) and partner-neutral trials (*N* = 6). The latter was extracted from the Emotional Movie Database (Carvalho et al., 2012). Blocks were displayed in a randomized order across participants and stimuli were displayed in a pseudo-randomized order across blocks.

Each trial consisted of: (1) fixation cross (5 s); (2) instructions in accordance with the current block (3 s); (3) video vignette (20 s); and (4) behavioral response (4 s).

**Figure 1:**
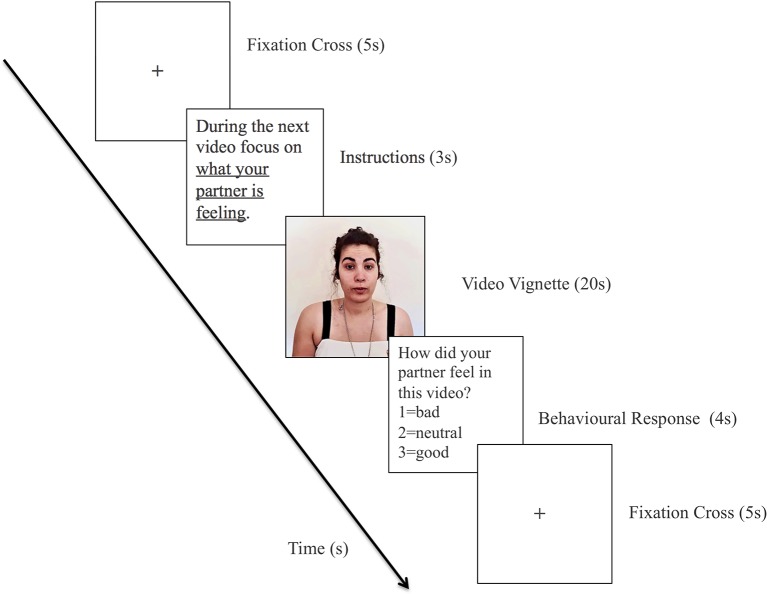
Scheme of an emotional trial for the other condition. To keep the confidentiality of the participants the image contained in this Figure corresponds to the photograph of the first author of this work who permitted its inclusion.

An example of a trial is shown in Figure 1. In the block *Other*, the performance was assessed in terms of the percentage of trials in which participants accurately detected their partner’s emotional state (e.g., by choosing a negative descriptor for vignettes in which partner expressed negative emotions or contents). In the block *Self*, since the participants were reporting their own emotion, performance was measured in terms of the percentage of trials in which the participant reported an emotional state congruent with that expressed by their partner. The results of the behavioral responses provided by the participants at the end of each trial can be found in Supplementary Table S1. However, it should be noted that these behavioral responses aimed essentially to ensure that the participants were focusing on their own (in self-condition) and the other’s experience (other conditions).

### Image Acquisition

Structural (T1) and functional images (T2*) were acquired with a clinically approved 3T MRI scanner (Siemens Magnetom Tim Trio, Erlangen, German). Data were acquired from each participant in a session that included one structural T1 scan [192 sagittal slices, repetition time (TR) = 2,000 ms.; echo time (TE) = 2.33 s, flip angle (FA) = 7°, slice thickness = 0.8 mm, slice gap = 0 mm, pixel size = 0.8 × 0.8 mm^2^, the field of view (FoV) = 256 mm] and one functional BOLD sensitive echo-planar imaging (EPI) sequence [39 axial slices; repetition time (TR) = 2,000 ms.; echo time (TE) = 29 ms., FA = 90°, matrix size = 64 × 64, slice thickness = 3 mm, pixel size = 3 × 3 mm^2^, field of view (FoV) = 222 × 222 mm]. Synchronization between the paradigm and the acquisition was insured for each TR.

### Data Preprocessing

Data preprocessing was performed using the Statistical Parametric Mapping software (SPM12; Wellcome Department of Cognitive Neurology, London, UK^1^). Preprocessing steps included: (1) slice-timing correction; (2) motion correction through the re-alignment to the mean image; (3) rigid-body registration of the mean functional image to the T1; (4) normalization of the functional acquisition to the Scale barr Neurological Institute (MNI) standard space (Ashburner and Friston, 1999) through the application of a rigid body transformation and a nonlinear spatial normalization following nonlinear registration of the T1 to the MNI T1 template; (5) regression of motion parameters, white matter (WM) and cerebrospinal fluid (CSF) signals; (6) smoothing with an 8-mm full-width half-maximum Gaussian kernel to decrease spatial noise; and (7) high pass temporal filtering (filter width of 128 s) to remove low-frequency noise. A general linear model (GLM) was inverted for each subject to identify subject-specific regional responses for subsequent DCM analysis. All images were inspected visually to ensure that participants had no brain lesions or disproportionate head motion. Nine participants were excluded: one due to head motion higher than 2 mm in translation and 1.5° in rotation; two due to anatomical abnormalities; two due to technical problems; four due to abnormal patterns of activation during the video condition.

### Effective Connectivity Analysis

In brief, our fMRI experimental design was a standard block design with a 2 × 3 factorial structure (a two-level *self* vs. *other* factors, and three levels of valence; *positive*, *negative* and *neutral*). For the DCM analyses, we focused on the main effects of the self-other factor. In other words, we asked whether this factor changed directed connectivity within the social brain network under study.

DCM was implemented using the Statistical Parametric Mapping software (SPM12; Wellcome Department of Cognitive Neurology, London, UK) to estimate the EC between the regions of interest (ROIs; Zeidman et al., 2019). Based on the group results from a previous study (Esménio et al., 2019), we selected the brain regions associated with high-level social processing (Bzdok et al., 2012; Schurz et al., 2014; Alcalá-López et al., 2018); namely, those regions activated during both self and other condition. These included the left and right MTG, the PCC/precuneus and the left and right TPJ. Since the RTPJ was the only significant region for the contrast self vs. others, this region was designated as the index node, which was connectivity dependent upon the self-other context.

Regarding the regional responses for each subject (i.e., the selection of the ROIs), five peak coordinates were used: LMTG (−56, −14, −12), RMTG (56, −10, −12), PCC/prec (−10, −52, 30), LTPJ (−49, −61 28) and RTPJ (50, −48, 18). These coordinates were obtained by combining several group analyses from the above-mentioned previous study (Esménio et al., 2019), and standard coordinates from the recent literature on the social brain (Alcalá-López et al., 2018). The regional responses corresponded to the principal eigenvariate within an 8-mm sphere centered on the corresponding subject-specific peak activation within 10 mm of the group coordinates.

**Figure 2:**
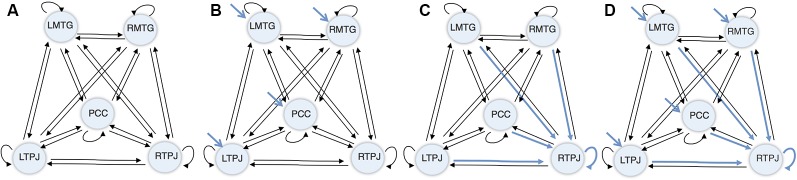
Dynamic causal modeling (DCM) initial model.** (A)** Connectivity architecture. **(B)** Driving inputs. **(C)** Modulatory effects. **(D)** Final model.

Next, a DCM model was computed for each subject, combining: (1) the five regions of interest; (2) a fully connected model (displayed in Figure 2A), where every region shares a connection with all the other regions in the network (i.e., full extrinsic connectivity) and has a connection to itself (i.e., intrinsic connections); (3) driving inputs on every node (apart from the index node—the RTPJ), where the driving input comprised the visual stimulation during the vignettes viewing blocks (displayed in Figure 2B); and (4) condition-specific or modulatory effects on all the afferents to the RTPJ (including intrinsic self-connections, as shown in Figure 2C).

In respect to the condition-specific or modulatory effect, it represented the effect of making inferences about a close other relative to self. Thus, based on previous findings showing rTPJ as the only region significant when inferring about Other rather than Self (Esménio et al., 2019), were selected as plausible locations all the connections to RTPJ, including the self-connection.

In summary, this five ROI model comprised a fully connected architecture (i.e., five intrinsic/self-connections and 20 between regions/extrinsic connections), four driving inputs exerting direct effects on four ROIs—the LMTG, the RMTG, the PCC, and the LTPJ—and five context-sensitive or modulatory effects (i.e., the four other ROIs that shared a connection with the RTPJ—LMTG, LTPJ, PCC and RMTG—and a self-modulatory effect). The ensuing model architecture is displayed in Figure 2D.

The selections of the position for the modulatory effect and the driving inputs’ locations were based on the results of the previous study (Esménio et al., 2019) and the connectivity architecture in Alcalá-López et al. (2018). The latest represents a meta-analysis that derives a social brain definition from 26 meta-analyses of social-cognitive capacities with significant convergence from original 25,339 initial foci from 3,972 neuroimaging studies in 22,712 participants.

After the model specification, this model was estimated and inverted for each subject, and the ensuing posterior densities over connectivity parameters (i.e., posterior means and covariances) were taken to the between-subject level for inference about group effects using Parametric Empirical Bayes (PEB; Friston et al., 2016).

A second level PEB model was then computed over the parameters to describe how group-level effects constrain parameter estimates on a subject basis. More specifically, the PEB model computed as second-level posteriors for each model the mean and differences of the group. These second-level posteriors were then used as empirical priors that shrink subject wise posterior estimates, thereby eliminating a between-subject degree of variability.

Accordingly, PEB random effects (RFX)’s underlying assumption is that all subjects use the same model architecture but express different parametric effects in terms of the connection strengths or their modulation. In other words, all subjects share the same architecture but express condition-specific effects to a greater or lesser extent.

The final step, the elimination of redundant parameters using BMR, enables one to identify context-sensitive changes in connectivity by comparing models that do and do not contain modulatory parameters.

BMR provides an efficient way to invert large numbers of reduced models, i.e., simplified versions of the full model that do not contain certain connections, following the computationally expensive inversion of a full model. These inverted models (full and reduced) are specified not only in terms of their priors but also their likelihood. The latter can then be used to identify which parameters/connections are redundant in the full model by comparing the likelihood of models that do and do not contain a certain connection.

**Figure 3:**
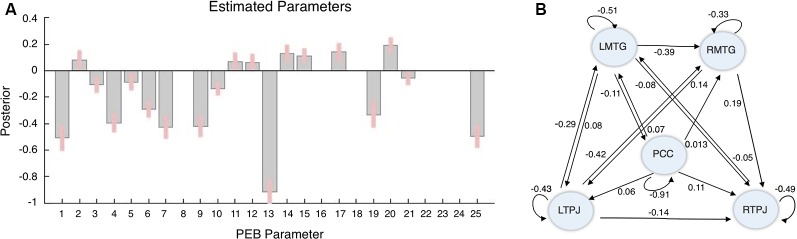
Average or “Baseline” Connectivity results. **(A)** Parameters posterior estimates. **(B)** The structure and parameters of the winning model. The black lines/values illustrate the (natural) connectivity between brain regions; i.e., irrespective of stimulus and task. The numbers are the strength of connectivity (Hz).

**Figure 4:**
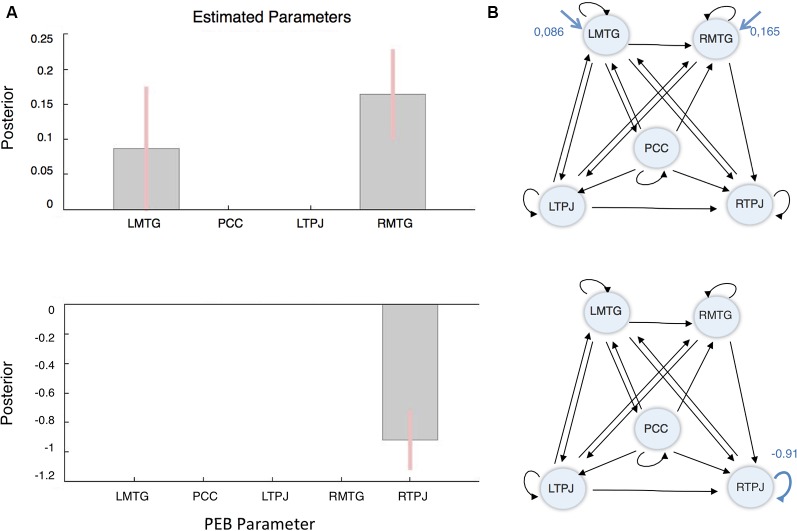
Driving inputs and modulatory effect results following Bayesian model reduction (BMR). **(A)** Driving inputs. **(B)** Modulatory effects or condition-specifics. (Left) Parameters posterior estimates (EP). (Right) The structure and parameters of the winning model. The black lines/values illustrate the connectivity between brain regions. The arrows in blue represent the driving inputs (upper) and modulatory effects (lower), respectively. The numbers quantify the strength of connectivity or information flow (Hz).

Three different PEBs (and consequent BMRs) were performed, each addressing one sort of connectivity: i.e., the average connectivity across conditions (corresponding to DCM’s matrix A); the driving inputs (matrix C—the blue arrows in Figure 2B); and the context-sensitive or modulatory effect (matrix B—the blue arrows in Figure 2C). The results of this reductive form of Bayesian model selection are shown in Figures 3, 4, where the parameter estimates of the extrinsic connections correspond to the underlying connection strengths or information flow (in Hertz). In respect to intrinsic connections, the effects are modeled in terms of the log scaling of inhibitory self-connection (of 0.5 Hz). Only connectivity parameters that survived to a posterior probability of 95% are shown (when comparing models with and without each parameter).

## Results

Regarding the connectivity architecture, a distinction must be made between the extrinsic and intrinsic connections. The extrinsic or directed connections reflect the EC between regions, i.e., the effect that one region has on another region (in Hertz), whereas the intrinsic or self-inhibition connections reflect how susceptible a region is to the influence of other regions. In other words, the self-inhibition connections reflect the rate of decay of neuronal activity in each region, where a lower self-inhibition means that a region is more sensitive to its inputs. Representing a rate of decay of neural activity the estimated parameters (EP) of intrinsic connections are log-scaling parameters set to default strength of −0.5Hz when the average connectivity across conditions is zero. Finally, while intrinsic disinhibition means a node increases sensitivity to all afferent inputs; an increase in extrinsic connectivity is specific to the afferent connection in question.

In terms of average connectivity several connections were removed: from LTPJ to PCC; from RMTG to LMTG and PCC; and from RTPJ to LTPJ, to PCC and RMTG. In the final connectivity architecture: (1) the PCC exerts a positive influence in every region but only receives input from LMTG; (2) the RTPJ receives input from all the other regions having only one efferent to LMTG; (3) the LTPJ exerts a negative influence on all the other nodes (except for PCC), which can be interpreted as a “tonic” inhibition (Stephan and Friston, 2010); and (4) in both bilateral regions; i.e., MTG and TPJ, the information flows from the left region to the right node. In respect of the intrinsic connections, we can see that there was a “tonic” negative self-inhibition in all the nodes in this network, particularly in the PCC (Ep = −0.91). The resulting connectivity architecture is summarized in Figure 3.

In terms of driving inputs, the winning model retained only driving inputs to the bilateral MTG (left, Ep = 0.086 Hz and right, Ep = 0.165 Hz). Finally, in terms of the context-sensitive changes in connectivity, the only modulatory effect that survived BMR was a decrease in RTPJ’s self-inhibition (Ep = −0.91). In other words, RTPJ was disinhibited during the other condition; thereby increasing its sensitivity to all its afferents. These results are summarized in Figure 4.

Finally, to take these results further, we performed a supplementary PEB analysis, focusing exclusively on the combination of intrinsic (within the region) connections (i.e., all intrinsic connections were allowed to change). The results show that the disinhibition in RTPJ’s (Ep = −0.64) sensitivity was accompanied by a decrease in LMTG’s (Ep = 0.22) intrinsic sensitivity. In other words, if all nodes are allowed to change their excitability, the differential activation elicited in RTPJ is explained by a reciprocal change in LMTG’s and RTPJ’s excitability. These results are shown in Supplementary Figure S1.

## Discussion

Previous research in social neuroscience of self and other processing speaks to the existence of shared neural systems for self and other processing (e.g., Decety and Sommerville, 2003; Lawrence et al., 2006; Lombardo et al., 2010; Rütgen et al., 2015; Lamm et al., 2016). In particular, in the context of romantic relationships, a remarkable overlap was found in the brain regions recruited when attending to one’s own internal states and those of a partner—as shown in our previous work (Esménio et al., 2019). This previous study also revealed that focusing on the partner preferentially recruited further brain regions involved in socio-cognitive processes, such as the RTPJ.

Hence, in the present study, we used DCM (combined with PEB and BMR) to estimate the information flow within a social brain network comprising the bilateral TPJ, PCC/precuneus and bilateral MTG, during a social inference task. We were especially interested in analyzing changes in directed connectivity or information flow when participants focused on their romantic partner, rather than on themselves.

Our results showed that—in terms of extrinsic connections—in the final model a small number of extrinsic connections were redundant, in particular the connections to the PCC and from the RTPJ. Regarding the PCC, these results suggest that—as in the Default Mode Network (DMN) which is also known as a mentalizing network—this region seems to have a coordination or orchestrating role within social brain networks (Hagmann et al., 2008; Deshpande et al., 2011; Raichle, 2015; Esménio et al., 2019). Also, based on findings of the analyses developed by Alcalá-López et al. (2018), using fMRI task-constrained and task-unconstrained modalities to compute the “functional coupling” between 36 social brain seeds, the PCC is a plausible candidate for mediating the information flow between low level-limbic networks and high-level cognitive networks devoted to social processes.

On the other hand, the RTPJ appears to play the role of a receptor node within the network under analysis, as it shares afferents connections with all the nodes, having only an efferent connection with the LMTG. This result is particularly interesting when considering the role of this region in high-level cognitive processes, such as detection of intention, belief reasoning, perspective-taking and self-other distinction (Brass et al., 2009; Santiesteban et al., 2012b; Ramsey et al., 2013).

Regarding the driving inputs—that correspond to the stimulation during the vignette blocks—we found that the only necessary driving inputs were those that entered through the bilateral MTG. Taking in consideration that the driving inputs were conveying visual and auditory information, this result goes in line with the study by Alcalá-López et al. (2018), that suggests that the lower sensory social networks are connected with high-level social neural systems through the bilateral MTG and the bilateral posterior superior sulcus (pSTS). Finally, this result suggests that the social information provided by the stimuli may have first entered the system through language and narrative processing regions (Spreng et al., 2009; Mar, 2011) to be represented and then assimilated hierarchically by more integrative or high-level regions, such as the PCC and the TPJ.

At last, concerning the modulatory effect of focusing on other relative to self, even though we tested for models where the RTPJ could selectively increase its sensitivity to different afferents or inputs, we found that a sufficient explanation for our data was an increase in postsynaptic responsiveness—as mediated by intrinsic disinhibition. It is generally thought that these changes in excitability rest upon fast synchronous interactions between inhibitory interneurons and pyramidal cells that express NMDA receptors (Moran et al., 2011, 2013; Symmonds et al., 2018). It is important to note however that in this DCM analysis, we do not identify the source of the neuromodulatory effects mediating the social process under study; we only identify the brain regions that constitute the targets of any context-sensitive modulation.

In summary, departing from the well-documented relationship between the selected regions, i.e., the PCC, TPJ and MTG, and social cognitive processing (Mar, 2011; Schilbach et al., 2012; Schurz et al., 2014), our results helped to characterize the different roles that each of these nodes may play within this social brain network. Regarding the PCC, similar to what has been found in resting-state studies of DMN connectivity (Hagmann et al., 2008; Deshpande et al., 2011; Raichle, 2015; Esménio et al., 2019), this region appears to play a central role within this network, by exerting an excitatory effect on all the other nodes. On the other hand, in this experimental paradigm, the bilateral MTG served as the entry point for stimulus bound driving input; suggesting that sensory information is first processed in a region that is associated with language and narrative comprehension (Spreng et al., 2009; Mar, 2011).

Finally, in line with findings that support a key role of the RTPJ in social cognition, particularly in self-other distinction processes (Saxe and Kanwisher, 2003; Aichhorn et al., 2006; Santiesteban et al., 2012a), our results showed that an increase in the RTPJ’s sensitivity to afferent inputs from other nodes was associated with the process of focusing on the romantic partner (rather than on the self). Since this region has been causally involved in differentiating self and other representations (Santiesteban et al., 2012a), a possible explanation is that to adopt their partner’s perspective, participants had to inhibit their perspective (Steinbeis, 2015). However, the role of the RTPJ in the specific dynamics of enhancing vs. inhibiting self-other representations remains unclear.

The results of this study endorse the importance of using EC analytic methods, such as DCM, which can estimate the effect that one neural system exerts over another, to understand the dynamic interplay between the nodes of complex brain networks. Technically, this sort of analysis allows one to quantify intrinsic (self) connectivity that transpired to play a crucial role in this study. This is important because functional connectivity measures (such as those afforded by correlations or Granger causality) preclude such characterizations. This is particularly relevant when studying high-level psychological phenomena such as social cognition that entail different subprocesses and recruit distinct brain regions.

As with all DCM studies, there are a few qualifications that should be borne in mind, when interpreting the functional architectures and estimates of EC. First, DCM and related approaches do not pretend to provide true or veridical estimates of directed neuronal coupling. The objective is to find the best explanation for the data in terms of simplified models of EC. In other words, the architectures—and condition or context-sensitive changes in coupling identified in our analyses—are the best explanations for the data, in terms of model evidence or marginal likelihood. This means that they provide the most parsimonious (i.e., simplest) and accurate account. This follows because log evidence is accuracy minus complexity, where complexity is scored by the divergence between posterior and prior estimates of the model parameters: minimizing complexity precludes overfitting and underwrites generalization to new data.

A second issue that deserves comment is the use of fMRI time-series. At first glance, it may seem implausible that differences in neuronal coupling—that rest upon fast neuronal transients, in the order of 100 ms—can be detected by fMRI. The reason why DCM works with slow hemodynamic responses is that changes in EC produce changes in the amplitude of fast evoked responses. After convolution with hemodynamics, these amplitude differences can be detected efficiently with fMRI. This is a key advantage of having a forward or generative model of how slow, hemodynamic responses are caused. In other words, the evidence for a model with changes in coupling—and accompanying fast neuronal responses—means that one can assess the evidence coupling changes, even if the fast neuronal responses cannot be observed directly.

Regarding future directions, it would be interesting to extend the present analysis and use DCM to characterize context sensitive changes in connectivity within other social brain networks (e.g., the empathy network; the putative mirror-neuron network). Another important contribution would be to study the dynamic interplay between different networks involved in different dimensions of social processing. For example, to use DCM to characterize the coupling between more embodied or affective systems and more cognitive or conceptual systems. These analyses would allow us to better understand the functional integration of affective and cognitive aspects of social processing. Specifically, it would help establish if these respective networks are hierarchically related—in a way that mental state attribution depends on the capacity to share another’s internal states. Finally, it would be interesting to examine the existence of similar EC patterns in other human dyads, such as parent-child or therapist-patient exchanges.

## Data Availability Statement

The raw data supporting the conclusions of this article will be made available by the authors, without undue reservation, to any qualified researcher.

## Ethics Statement

The studies involving human participants were reviewed and approved by University of Minho Institutional Review Board. The patients/participants provided their written informed consent to participate in this study.

## Author Contributions

JC and ÓG designed the study concept and design. JC and PO-S collected data for the experiments. SE performed the data analysis, interpretation and wrote the article under the supervision of JC, JS, and KF. All authors reviewed and approved the final draft.

## Conflict of Interest

The authors declare that the research was conducted in the absence of any commercial or financial relationships that could be construed as a potential conflict of interest.

## Footnotes

^1^http://www.fil.ion.ucl.ac.uk
