# Supplementary material for: Changes in the Effective Connectivity of the Social Brain When Making Inferences About Close Others vs. the Self
Source: Front Hum Neurosci. 2020 Apr 29;14:151. doi: 10.3389/fnhum.2020.00151 (PMC7202326; doi:10.3389/fnhum.2020.00151)
Supplement: Supplementary file 1 [file Table_1.DOCX]

**Supplementary Material**

Supplementary Table 1. Results of behavioral responses provided by the participants at the end of each trial. In the block *Other,* performance was assessed in terms of the percentage of trials in which participants accurately detected their partner’s emotional state (e.g. by choosing a negative descriptor for vignettes in which partner expressed negative emotions or contents). In the block *Self,* since the participants were reporting their own emotion, performance was measured in terms of the percentage of trials in which the participant reported an emotional state congruent with that expressed by their partner. Please note that these behavioral responses aimed essentially to ensure that the participants were actually focusing on their own (in self-condition) and on the other´s experience (other condition).

| Other | | Self | |
| --- | --- | --- | --- |
| Accuracy (%) | **Frequency** | **Congruency (%)** | **Frequency** |
| 100 | 10 | 100 | 13 |
| 99 – 75 | 16 | 99 – 75 | 12 |
| 74 - 50 | 2 | 74 – 50 | 1 |
| 49 - 25 | 0 | 49 - 25 | 2 |
| < 25 | 0 | < 25 | 0 |

Supplementary Figure 1. Results of specifying a modulatory effect in every intrinsic (self-) connection. (a) Parameters posterior estimates (EPs). (b) Connectivity matrix. (c) Structure and parameters. The black lines illustrate the baseline connectivity between brain regions. The blue arrows and values represent the modulatory effects and respective strength of connectivity (Hz).


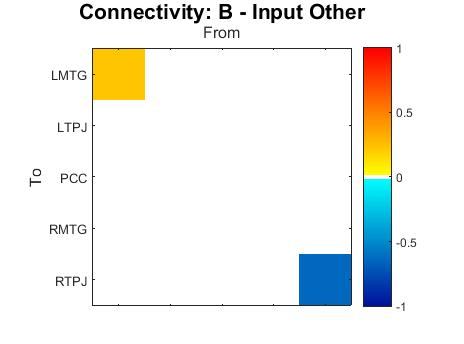


From

To

LMTG

LTPJ

PCC

RMTG

RTPJ

LMTG LTPJ PCC RMTG RTPJ

1

0.5

0

-0.5

-1

a)


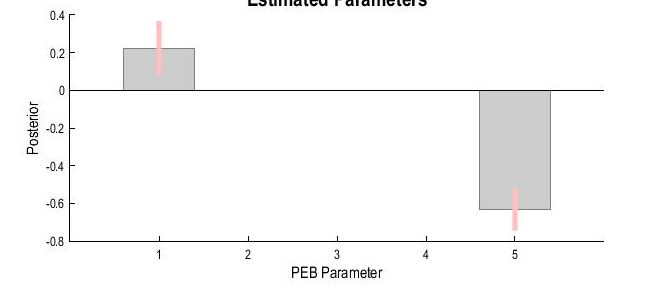


Estimated Parameters

0.4

0.2

0

-0.2

-0.4

-0.6

-0.8

PEB parameters

1 2 3 4 5

b)

c)

0.22

LMTG

PCC

LTPJ

RTPJ

RMTG

-0.64
